# Academic affairs and global health: how global health electives can accelerate progress towards ACGME milestones

**DOI:** 10.1186/s12245-015-0093-0

**Published:** 2015-12-02

**Authors:** Alison Schroth Hayward, Gabrielle A. Jacquet, Tracy Sanson, Hani Mowafi, Bhakti Hansoti

**Affiliations:** Department of Emergency Medicine, Yale School of Medicine, New Haven, CT USA; Department of Emergency Medicine, Boston Medical Center, Boston, MA USA; Department of Emergency Medicine, University of South Florida, Tampa, FL USA; Department of Emergency Medicine, Johns Hopkins School of Medicine, Baltimore, MD USA

**Keywords:** Global health, Emergency medicine, Graduate medical education, Competency-based education

## Abstract

Global health electives (GHEs) have become a standard offering in many residency programs. Residency electives should aid residents in achieving outcomes in the Accreditation Council for Graduate Medical Education (ACGME) competency domains. In this paper, the authors review existing literature and provide expert opinion to highlight how global health electives can complement traditional training programs to assist residents in achieving ACGME milestones, using emergency medicine residency as an example. Recommendations are provided for identifying exemplary global health electives and for the development of institutional global health elective curricula in order to facilitate milestone achievement. Global health electives can advance progress towards ACGME milestones; however, they may vary greatly in terms of potential for learner advancement. Electives should thus be rigorously vetted to ensure they meet standards that will facilitate this process. Given that milestones are a newly introduced tool for assessing resident educational achievement, very little research is available currently to directly determine impacts, and further study will be needed.

## Review

### Introduction

The number of physicians-in-training participating in global health electives (GHEs) is continually increasing. The proportion of medical students who report participating in GH experiences has increased annually from 27.2 % in 2006 to 30.8 % in 2010 [1]. A 2008 review of the websites of the 129 accredited medical schools found that nearly half had established initiatives, institutes, centers, or offices for global health [2]. There has been a concomitant rise in residents engaging in GHEs as part of graduate medical training. Recent surveys of residency directors have found that 70–91 % of residency programs offer international elective rotations [3, 4], and national surveys of residents have shown that they view the availability of an international elective as a positive factor in ranking residency programs [5].

While many educators agree that GHEs can provide numerous personal as well as educational benefits [2, 6, 7], there is little consensus on how best to design them to realize these benefits most productively and to formally align GHEs with the priorities for graduate medical education [8].

**Fig. 1:**
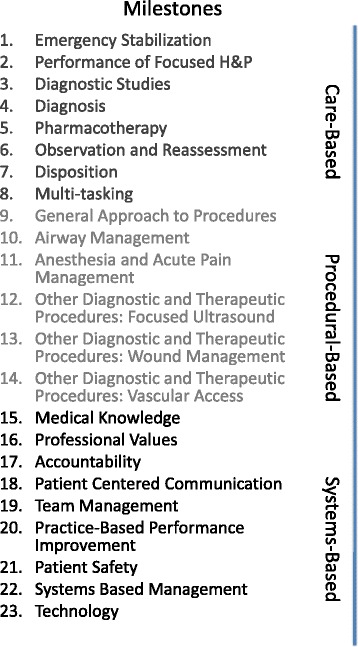
Emergency medicine residency milestones

In 1999, the Accreditation Council for Graduate Medical Education (ACGME) designated six core competencies that serve as broad domains to direct resident education [9]. The six competencies are as follows: patient care, medical knowledge, practice-based learning and improvement, interpersonal and communication skills, professionalism, and systems-based practice. Recently, there has been a move to define attainment of these competencies more specifically with the development of competency-based standards of achievement or milestones [10]. The milestones are specialty-specific, developed by members of each specialty [10]. Each milestone is tied to one of the previously defined core competencies—the milestones for emergency medicine residency are provided for illustrative purposes (Fig. 1) [11]. Each milestone has five defined performance levels by which a resident’s skill can be measured, where level 4 is designated as the residency graduation target.

In this paper, we highlight how well-designed global health electives can help residents achieve milestones, and describe optimal characteristics of GHEs, to assist in the development of specific electives that will facilitate milestone achievement.

### Patient care

The majority (14/23) of emergency medicine residency milestones address patient care. Global health electives provide a rich environment for trainees to enhance their patient care skills and to hone their history-taking and physical examination skills (Fig. 1, Milestones # (M#)2, 4) when their access to high-cost diagnostics is limited. Significant constraint on the use of diagnostic testing exists in most of the rest of the world: in high-income countries with publicly funded healthcare systems, or low-income countries where the cost of such diagnostics falls directly to patients who may not be able to afford them (or where they are simply not available). Skyrocketing healthcare costs in the USA have led to a renewed emphasis on cost effectiveness [12]. Judicious utilization of high-cost diagnostics will be highly relevant to trainees’ future practice. Learning this skill supports M#3, 4, 7, and 22.

In the USA, the ancillary staff perform most basic procedures such as establishing intravenous access, performing electrocardiograms, inserting urinary catheters, and uncomplicated wound management. GHEs provide residents ample opportunity to improve proficiency with such skills where ancillary resources are more limited (Fig. 1, M#13, 14). In addition, in many international settings, generalist physicians have a wider scope of practice. Residents engaging in GHEs in such settings may have the ability to observe or assist in procedures that they would encounter less frequently at their home institutions, such as cricothyroidotomy, thoracotomy, or even emergency craniotomy (Fig. 1, M#9, 10, 13, 14). Residents should be strongly discouraged from performing procedures independently that they have not been certified to perform in the USA. When properly supervised by experienced providers, GHEs can be a catalyst for rapid attainment of procedural competency.

Appropriate supervision is particularly important given that trainees may perform procedures or make diagnostic decisions without the same level of specialist support they have at their home institution. Navigating these situations is an important learning opportunity for trainees and simulates the transition many face when commencing practice in a smaller community hospital environment.

### Medical knowledge

The single milestone under the medical knowledge competency (Fig. 1, M#15) is measured by trainees’ performance on standardized tests, such as the in-training examination and the American Board of Emergency Medicine (ABEM) qualifying examination. The ABEM exams are based on the Model of the Clinical Practice of Emergency Medicine (EM Model) [13]. A review of this document reveals numerous topics which trainees are likely to encounter more frequently on GHEs. These include the following: toxicologic emergencies (pesticide exposure, caustic ingestions, bites stings, and envenomations), acute infectious emergencies (e.g., acute hepatitis, parasitosis, mycobacterial infections, malaria, and acute HIV), and long-term sequelae of infectious diseases now rarely seen in the USA (e.g., rheumatic fever and mitral stenosis from RF, tertiary syphilis), among others.

Easy access to low-cost, long distance travel has made infectious disease from distant parts of the world more relevant to US physicians. This has been evidenced recently by the rapid spread of pandemic influenza and the deadly MERS virus, a zoonosis originating in a camel reservoir in the Arabian peninsula that resulted in deaths here at home in Indiana [7]. The opportunity to see and treat patients with even routine tropical illnesses (e.g., malaria) that are rare in the USA will better prepare physicians to treat such patients at home. To maximize the opportunity for expanding their fund of knowledge, residents should receive pre-departure training in local disease entities and the functioning of the local health system prior to beginning their practice on a GH rotation, with qualitative assessments at the conclusion of the course to ensure learning objectives are achieved [6].

Medical knowledge is the competency area encompassing the broad fields of epidemiology and public health. In addition to the experience of medically managing conditions and presentations that are rare in the USA, GHEs in low-resource environments can further help residents learn about underlying socioeconomic and political determinants of health and to understand how inequities impact survival and health access—lessons that apply to working with marginalized populations at home (Fig. 1, M#16, 18, 22). With different cultural, social, and economic constraints, common medical and surgical complaints become new challenges [14].

### Professional values

To demonstrate this competency (Fig. 1, M#20), residents must demonstrate a commitment to carrying out professional responsibilities, an adherence to ethical principles, and a sensitivity to diverse patient populations. Global health electives provide opportunities for residents to demonstrate level 3 mastery of the professional values milestone, M#16—the recognition of ways in which their personal beliefs impact the care they provide and management of those beliefs in order to optimize relationships and medical care. Managing personal values and beliefs may be especially difficult when confronted with common ethical dilemmas that accompany working in a setting with limited resources or with the experience of very different cultural or ethical norms.

Attitudes about issues related to health and well-being are based on specific choices made by each culture and priorities placed on various problems in society. As such, residents may encounter situations where the accepted norms they encounter on a GHE may conflict with their personal ethics or accepted norms in their home communities. Residents used to universal access to emergency care, for example, may be surprised to see such care denied to patients in a hospital until they or their family can pay for treatment up front. Such situations may reflect that society’s decisions about resource allocation in an attempt to provide more basic services (e.g., vaccination) to the greatest number of people.

There is a danger of residents returning from GHEs feeling scarred by experiences where they either observed or participated in clinical activities that they did not agree with and were not equipped to handle. Residents should be protected from violating their ethical codes of conduct and properly designed GHEs should provide them with a framework for how to deal with such conflict. Various methods may be utilized including focused discussions, case-based learning, and assigned readings. These preparatory steps can help residents recognize ethical conflicts and to envision how they might engage with their local colleagues to reach a solution that promotes the health and well-being of their patients, respects their host culture, and does not violate their personal ethics. Experiencing such challenges on GHEs and being equipped to work through them can make residents better trained to negotiate such conflicts at home and give them a broader understanding of the cultural factors that inform their patient’s health choices.

The GH elective may be a particularly useful opportunity to determine whether a trainee recognizes the limits of his or her knowledge and can maneuver through ethical dilemmas in uncommon or particularly challenging clinical situations, which is a level 4 achievement in the professional values milestone, expected to be achieved by graduation from residency. This type of situational learning additionally advances the trainee towards high-level attainment in the accountability milestone (Fig. 1, M#17), in regard to awareness of knowledge limits.

### Interpersonal and communication skills

To demonstrate this competency residents must display effective interpersonal and communication skills, both with professional colleagues and with patients and their families. Learning cross-cultural differences in communication and how to adapt one’s style of communication to widely varying situations will improve trainees’ ability to inform patients about their condition and the treatments available to them (Fig. 1, M#2, 16, 18). GHEs support the development of such communication skills as residents encounter patients who are often quite different from themselves in terms of background. For some trainees, it can represent the first time that they are a racial minority in the community with which they are interacting. In order to assure that residents participating in GH electives are able to develop these skills, it is crucial that the clinical setting be adequately staffed, with a mentor who is able to speak fluently in a language in which the resident also is fluent and who has adequate time to discuss patient cases and to directly observe a number of patient encounters.

GHEs can help trainees achieve at least a level 3 proficiency in the patient-centered communication milestone (Fig. 1, M#18), for effective communication with vulnerable populations. Utilization of flexible communication strategies to deal with challenging patient populations and development of conflict management skills demonstrate progress towards level 4 or 5. This milestone can be assessed by direct observation of patient encounters by the trainee’s mentor. Skills in cultural sensitivity might be reinforced by structured discussion groups or symposia with other participants in GH work, which may be organized with the assistance of a medical school office of global health.

The 2010 US Census demonstrated that 9 % of US residents over the age of 4 years had limited English proficiency. The inability to communicate in a patient’s own language and the use of ad hoc interpreters have been shown to have adverse clinical consequences [15], to result in communication errors in medical care, and to hamper the ability to properly obtain informed consent for treatment and research [16]. GHEs may increase residents’ foreign language proficiency and thereby improve communication skills for use in their clinical practice back home (Fig. 1, M#18, 20).

Effective communication is often dependent on the appropriate utilization of interpreters. Prior to embarking on a GH experience in a location with a foreign language in which they are not fluent, residents can add to their skill set by completing formalized training on the effective use of trained medical interpreters [17]. Programs may utilize shadowing medical interpreters at home institutions or simulated interviews with medical interpreters as a means of either training residents or to assess their competency and improvement in communication in a foreign language.

### Practice-based learning and improvement

GH rotations offer an opportunity to familiarize trainees with standardized guidelines set out by the World Health Organization (WHO). For example, the strategies of Integrated Management of Childhood Illness (IMCI) and Adult Illness (IMAI) [18, 19] highlight the diagnosis and treatment of conditions common to developing countries, and these can be supplemented by local clinical practice guidelines.

Adaptation of evidence-based medicine (EBM) to resource-poor settings is an important component of a GH curriculum [20]. Residents should consider the evidence behind alternative therapies used to control costs in such settings, e.g., use of streptokinase vs. recombinant tissue plasminogen activator (tPA) for thrombolysis in acute coronary syndrome and stroke. Critically evaluating and implementing different practice patterns will increase trainees’ versatility and ability to adapt their individual practice to match their practice environment to maximize patient outcomes (Fig. 1, M#20).

Residents working in resource-limited settings can learn unique practice skills such as how to strategically elect to avoid intubation in certain patients when there are limited ventilators available or how to manage a patient in respiratory failure when there is no ventilator available. These kinds of ethical decision-making and resource-utilization strategies are rarely taught in the USA but could be valuable for community disaster preparedness planning. Making such difficult decisions advances competency towards the professional values milestone (Fig. 1, M#16) at the level 4 or 5 standard [14].

As ambassadors of the specialty of emergency medicine, residents may provide teaching to international colleagues while on rotations, for example, by supervising trainees from the local medical school or residency program. This is particularly true in countries where emergency medicine has only recently been recognized as a specialty in its own right, and role models in the specialty are fewer in number. Proficiency at teaching evidence-based medical knowledge is a high-level skill (level 5) in the practice-based performance improvement milestone (Fig. 1, M#20).

### Systems-based management

Exposure to public health organizations, policy, and advocacy issues can play a significant role in building the resident’s understanding of systems-based practice not only in the international setting but also in the USA. Key components include learning about different healthcare systems and fee structures and understanding their impact on access to care and quality of care. Residents will have the opportunity to gain a heightened sensitivity to the implications of systems issues not only to the individual patient but also globally to the healthcare industry.

In many countries, emergency medicine is developing as a specialty faster than the emergency services infrastructure. Residents rotating on GHEs in such countries may discover that even with the best EM knowledge and skills, their ability to meet patients’ needs is significantly constrained if access to services before (e.g., emergency transport) and after the emergency care encounter (e.g., surgery, intensive care) is limited. Such experiences provide a foundation for understanding the importance of infrastructure in the provision of high-quality medical care (Fig. 1, M#7, 22). Based on observation of the trainee’s comprehension in this area, a GH elective may help him/her reach level 3 or potentially level 4 competency in the systems-based management milestone.

Practicing in a clinical environment as an invited guest requires a careful balancing act. Specifically, it is often difficult to balance the impulse to implement previously learned protocols for care with the willingness to learn from international colleagues about their practice and standards. Dealing with this challenge can help trainees understand that a systems-based practice cannot be transplanted into a new environment and must be tailored to the community it serves (Fig. 1, M#22). Additional benefit might be gained from structured opportunities to discuss the healthcare system with local clinicians or other trainees in-country or participation in projects for the improvement of clinical operations while on elective.

## Conclusions

Global health electives enhance medical training and professional development in a variety of ways that are beneficial to patients and practitioners back at home. Work remains to develop structured evaluations of GHEs that specifically assess how well they meet ACGME competencies and support graduate medical education through achieving specific educational milestones. Efforts should be made to integrate definitive skills that can be gleaned from global health electives into future iterations of the ACGME milestones. Defining educational targets of GHEs using the same competencies used to define training at home institutions will help to standardize—and maximize—the educational benefits that these unique and life-changing experiences can offer.
